# Fulton County Medical Society

**Published:** 1914-03

**Authors:** R. R. Daly


### FULTON COUNTY MEDICAL SOCIETY.

### REGULAR MEETING THURSDAY, FEBRUARY 19, 1914.

R. R. Daly M. D.

Dr. L. Sage Hardin exhibited a case of large Cirsoid Angioma of the right orbit. Its duration has been several years during which time it has grown so as to obstruct the vision on that side by its very size. The other eye has very poor vision from other causes and it was imperative that this tumor should be treated and if possible, removed. The right carotid had been ligated when he was aged 15, but with no good result. There has been marked pulsation for a long time since then. Last October, Hardin began Wythe's injection of boiling water and made three treatments of this character. On the first of January and again on the first of February, he gave deep injections of alcohol. There was no pain after the boiling water, but pain and haemorrhage followed the injection of alcohol. The tumor is now largely fibroid in character as a result of the boiling and the destructive effect of the alcohol. It is less than half its former size and the expectation is to remove it in safety soon.

Dr. E. G. Jones presented a case that had recovered from what has appeared to be a Carcinoma of the Stomach. The man is 55 and said that all his life he had suffered with indigestion and various pains in the stomach and abdomen. He was reduced to less than a hundred pounds in weight and expected to die. Last November, he was vomiting 12 and 24 hour material. but he felt more comfortable for an hour when the stomach was full. X-ray showed prolapsed stomach with six hour residue and evidences of great pyloric obstruction. He was put on rectal nourishment and then operated upon. A large tumor presented at the pylorous that had all the appearances of a carcinoma. A gastro-enterostomy was done and he improved so rapidly that in 16 days he was ready for the secondary operation and it was performed. But instead of finding a carcinoma, the major part of the tumor had disappeared and the work was a greatly simplifyed resection. The man spoke for himself and said that he had never felt so well and that he weighed 155 lbs.

Dr. Derr said that the radiograph he took at the time, showed dilatation, ptosis and pyloric obstruction. He displayed other pictures of 6 hour residues in cases that had required operation. He said that a horn-shaped stomach contraindicated operation.

Dr. Niles said that any stomach showing 7 hour residue is an operative one and that all cases are temporizing with fate when pyloric obstruction is shown by 24 hour rejection. The main lesson is of optimistic medication and surgery. No one can act as the supreme court because dangerous appearing cases sometimes get well when scientifically treated.

Dr. L. L. Andrews said that it was marvellous the large amounts of material that were removed from the stomach of Dr. Jones' case. He had been associated in its care. He told in addition of his own father who was 71 when he was operated upon for ptosis of the stomach and recovered his health and strength so that he was happier than he had been in many years.

Dr. Hardin said that if these cases were taken early when the ulcers were small, great distress could be saved. He gave credit to Dr. Winn for making many such pictures for him.

Dr. S. R. Roberts showed a boy who had osteo-myelitis of the left inferior maxilla several years ago. It apparently recovered completely. On January 21, he was kicked by an unshod colt in the right supra orbital plate. The cut healed promptly and he went to school. On the 26th he had soreness and pains and the next day, he cried at table because he could not get his mouth open. On the 28th, the old wound opened and showed pus. There was severe lock jaw, and other symptoms showed tetanus. The wound was cleansed with iodine and anti-tentanic serum was given, 10,000 in the vein of the arm, 3,000 intra spinous and 3,000 around the wound. Double urticarial eruption immediately followed the vein injection. The temperature rose to 104.6 and there was opisthotonos. On the 30th there were other injections of 10,000 in spine and vein. Bacilli were found in the wound made at the spinal injection. He was given altogether 48,000 units. On February 14, he left the hospital. He can not open his mouth so very well as yet, but he can eat. In Robert's opinion one should force the serum in all these cases and judge by results. If this is done promptly, the mortality will fall from 80 or 90 per cent. to 10 or 20%.

Dr. Thrash said it was interesting because it showed that the bacilli travel instead of the toxines. They pass along the nerves and it would be good practice to inject the nerves themselves whenever possible. The minor nerves might be cut and thus the path of the infection interrupted.

Dr. Wagnon called attention to the fact that urticaria such as showed in this case often came also with the injection of Salvarsan.

Dr. Roberts said that the serum is very expensive and that $60.00 was spent in this case. Often it is delayed on that account, but it should be used as soon as the diagnosis is made. It was interesting to note that the character of the wound, having been made by a hoof, was just such as would introduce the tetanus germ.

Dr. E. C. Thrash read a paper "Tuberculosis an Ingestion Disease." His thesis goes to show that the germs are swallowed and reach the circulation by means of the villi and that eventually they come to rest in some areas where the circulation is terminal. They can not develop unless they are at rest, and the breathing of them in does not give them that opportunity.

Dr. Niles called attention to the destructive quality of free hydrochloric acid in the stomach upon the organisms.

Dr. Funke said that the organisms will pass through the mucosae and that they might well get into the lung tissue if breathed into the bronchioles. They are found in the blood at times, and Guinea Pigs have been infected through blood injections. One can not rule out the aerogenous method of infection in his opinion. Moreover, tuberculosis of the brain is rare and here in the cortex, all the circulation is terminal.

Dr. Thrash said that so far as the germs getting through the stomach is concerned, the stomach was not always normal by any means and that free hydochloric acid was not always present. He showed also that the chief location of tuberculosis in the kidney was in relation to its terminal blood vessels.

Dr. R. M. Nelson read a paper on "Rarity of Sarcoma in the Sclera."

Dr. Daly said that the cases of malignant growth in the eye that had come to his experience had not been of this character. The melano sarcomata such as this simulated for a time, were attached to the choroid and retina and developed entirely within the eyes. These were extremely likely to recur and the metasis, being through the blood was likely to show the disease almost anywhwere when the tumor was operated.

Dr. Lokey showed two specimens, one a choridal melano sarcoma and the other partly within and partly without the eye ball. It was hard to say where it originated. There was recurrence in both cases and if it were to be done again he would perform a complete exenteration of the orbital cavity.

Dr. Stirling said that several years ago he examined the histories of 55 years' work in a large hospital in London and found that there were but 25 cases of primary sarcoma of the orbit and none of the sclera. He agreed with Lokey as to complete operation. He asked Nelson if he were sure of his pathologist and whether the tumor might not have come from the uvea.

Dr. Nelson said that there was no doubt as to the diagnosis or the skill of the pathologist.

REGULAR MEETING THURSDAY, MARCH 5, 1914.

Dr. E. C. Davis read a paper on "Who Should Practise Surgery?" It appears elsewhere in the Journal.

Dr. W. A. Crowe said that he concurred with the outlines as stated in the paper as to the requirements of a surgeon. The first and great essential of the surgeon was to be a good doctor. Each case should be studied in all ways and there is no real success in separating the medical and surgical powers.

Surgery isn't all cutting and stitching; it is knowing when to cut and when not to cut. Yet a man must not be timorous. Ideal cases are not always practical ones. A man should not try to do too much at one time. In fulminating cases of appendicitis, for example, it is enough to open and drain without trying at that time to take out all offending material in the abdomen. A great deal of judgment and consideration is necessary to the full equipment of the good surgeon and this all should be available quickly. That is where experience has its great value. In Hospitals, a Resident Surgeon is of the utmost value. He is skilled and at hand.

Dr. J. C. White said he agreed with the paper, but after all the experience a man could acquire, he might still not be a surgeon. Surgeons are born and they will do surgery no matter where you put them. They will do it well and equip themselves if there is no one to instruct them and they will take the greatest advantage of every opportunity to learn when instruction is at hand. But with all this the best surgeon is the one who can give his whole time to it and not spend energy in the practice of medicine. There should be a co-operation between the various workers so that the surgeon can specialize.

Dr. Davis closed by saying that the fee splitting evil was dangerous and disreputable and should be denied at every appearance of a demand. The induction of abortion is unduly frequent and surgeons have this to look to. When studying a case it is well to remember that it is always easy to get into an abdomen but not always possible to get out successfully.

Dr. J. L. Campbell read a paper on "Appendiceal Abscesses."

Dr. L. Sage Hardin said that retro-coecal abscess was often followed by sub-phrenic abscess with danger of septic emboli in the lung. The intra-peritoneal abscess often simulates typhoid fever.

The infection always begins inside the appendix and should be recognized early even in fulminating cases. When the peritoneum becomes involved, operation should be urged, but if this is not agreed to, rectal feeding will sometimes stop it. There is grave danger in giving cathartics to these cases and cathartics should not always be resorted to when there is trouble in that region. When there is abscess, the sero fibrinous exudate that is accumulating about the abscess to wall it off is swept away by the purgative and the protection destroyed.

An abscess should not be opened every time you see one. If it has been of several days' standing, give it a chance to wall itself off until it can be opened in safety. If it has been present 10 days and seems progressive, it should be opened without further delay.

Dr. Goldsmith said that he wanted to correct some of the statistics as they appeared in the paper concerning Grady Hospital. The percentage of mortality in these cases is too high when put at 22. The classification has been wrong and suppurative appendicitis and peritonitis must have been included. He agrees with Hardin in not hurrying to get into such an abscess before there was some protective walling off.

The ice bag, Fowler's position Oxchsner's treatment, etc., will all help to hold patient in good condition until the abscess is ripe and safe to open. The drainage should not be by a big tube partially removed from day to day, but by cigarette drains as many as are necessary and by counter openings in the flank. Post operative treatment must be careful. No drain should ever be lost.

Dr. M. T. Davis asked what was the effect of ether lavage in such cases.

Dr. Campbell closed and said that the Grady statistics were taken from the published reports and that he would be glad to know there was a lower mortality there than the reports stated. As to time of operation, he always follows the rule that when he has an abscess with signs of absorption he doesn't wait an hour. The average duration of his cases prior to operation was two weeks. One case was four days and was an emergency. He doesn't know anything about the ether lavage.

Dr. L. M. Gaines reported cases of Atropin Poisoning, Veronal Poisoning and General Paresis.

Dr. Lokey said that he had two cases of Atropin Poisoning. In one there was great depression and in the other there was muscular spasm.

Dr. Brawner said that he had three cases of staggering gait after Veronal and the doses were not very large in any of them.

He has used salvarsanized serum in general paresis with success and has cases under that treatment now in his Sanitorium.

Dr. Andrew Quillian related a case that had taken a teaspoonful of a 1% solution of atropin by mistake in the hospital. The stomach pump was quickly used. The delirum was mild but there was pain in the bladder and in a short time 72 ounces of urine were withdrawn. The case recovered.

Dr. Hardin said that veronal was dangerous in old people and that some deaths from it have occurred here.

